# The ClpP activator ONC‐212 (TR‐31) inhibits BCL2 and B‐cell receptor signaling in CLL

**DOI:** 10.1002/jha2.160

**Published:** 2021-01-14

**Authors:** Narjis Fatima, Yandong Shen, Kyle Crassini, Edwin J. Iwanowicz, Henk Lang, Donald S. Karanewsky, Richard I. Christopherson, Stephen P. Mulligan, Oliver G. Best

**Affiliations:** ^1^ Kolling Institute of Medical Research Royal North Shore Hospital University of Sydney Sydney Australia; ^2^ School of Life and Environmental Sciences University of Sydney Sydney Australia; ^3^ Madera Therapeutics LLC Cary North Carolina; ^4^ Department of Molecular Medicine and Genetics Flinders Health and Medical Research Institute (FHMRI) College of Medicine and Public Health Flinders University Adelaide Australia

**Keywords:** chronic lymphocytic leukemia, imipridone, TR‐compounds, tumour microenvironment

## Abstract

Despite advances in therapy, a significant proportion of patients with chronic lymphocytic leukemia (CLL) relapse with drug resistant disease. Novel treatment approaches are required, particularly for high risk disease.

The imipridones represent a new class of cancer therapy that has been investigated in pre‐clinical and clinical trials against a range of different cancers. We investigated the effects of the imipridone, ONC‐212, against CLL cells cultured under conditions that mimic aspects of the tumour microenvironment and a *TP53*ko CLL cell line (OSU‐CLL‐*TP53*ko). ONC‐212 induced dose‐dependent apoptosis, cell cycle arrest and reduced the migration of CLL cells in vitro, including cells from patients with TP53 lesions and OSU‐CLL‐*TP53*ko cells. The effects of ONC‐212 were associated with protein changes consistent with activation of the mitochondrial protease, CIpP, and the integrated stress response. We also observed inhibition of pathways downstream of the B‐cell receptor (BCR) (AKT and MAPK‐ERK1/2) and a pro‐apoptotic shift in the balance of proteins of the BCL2 family of proteins (BCL2, MCL1, BCLxL, BAX and NOXA).

In conclusion, the study suggests ONC‐212 may represent an effective treatment for high risk CLL disease by inhibiting multiple facets of the BCR signaling pathway and the pro‐survival effects of the BCL2‐family proteins.

## 1 INTRODUCTION

Chronic lymphocytic leukemia (CLL) is the most common form of leukemia in western countries and is associated with accumulation of CD5^+^ B‐lymphocytes, in the blood, bone marrow and lymph nodes.

Lymph nodes and bone marrow play significant roles in the pathogenesis of CLL. Leukemic cells infiltrate the lymph nodes and interact with stromal and other immune cells, forming pseudofollicular structures known as proliferation centres. These interactions and a range of cytokines and growth factors support CLL‐cell survival and promote their proliferation in the tumour microenvironment (TME).

The survival of CLL cells and the pro‐survival effects of the TME are dependent on signaling via the B‐cell receptor (BCR) and several key intracellular signaling pathways. This is highlighted by the efficacy of inhibitors of BCR signaling, including ibrutinib and idelalisib and the BCL2 inhibitor, venetoclax. Despite high response rates, relapse and drug resistance among patients treated with these novel agents is common. Identification of novel treatment approaches remains an important focus of research in CLL.

The imipridones are a novel class of anti‐cancer compounds that have shown activity against a variety of solid and haematological malignancies [1, 2, 3, 4]. Pre‐clinical studies of ONC‐201 [5] have led to several on‐going clinical trials in a range of solid tumours, and leukemias [6, 7, 8]. ONC‐212 (TR‐31) is a next‐generation imipridone that has been reported to have more potent anti‐cancer properties than the related compound, ONC‐201 [9, 10]. The mechanisms of action of ONC‐212 include activation of the mitochondrial caseinolytic protease (CIpP) [11, 12]. ClpP plays a critical role in mitochondrial protein homeostasis and is commonly over‐expressed in malignant cells, including in haematological malignancies [13]. Studies show that both inhibition and activation of ClpP activity may represent therapeutic strategies for this disease [12, 13].

There is also evidence that select imipridones also block cell signaling pathways associated with specific G‐protein coupled receptors [10] and inhibit components of the unfolded protein response (UPR) [9]. The proliferation of tumour cells often occurs under conditions that induce stress on the endoplasmic reticulum (ER) and activate the UPR [14, 15, 16].

Under normal homeostatic conditions, the ER chaperone, glucose‐regulated protein 78 (Grp78), binds to and decreases activity of the ‘stress‐sensors’ IRE‐1, PERK and ATF‐6. However, under conditions that induce ER stress, Grp78 dissociates from these sensors and initiates the degradation of misfolded proteins to mitigate cellular damage. During periods of prolonged ER stress, these adaptive responses can also initiate apoptosis [17, 18].

There is strong evidence to suggest that the UPR also plays an important role in the survival and proliferation of CLL cells [19]. ER stress is known to induce BAX/BAK mediated apoptosis [20, 21]. This is mediated, at least in part, through activation of ATF4 in the protein kinase R‐like ER kinase (PERK) arm of the UPR, which induces expression of the transcription factor CHOP [22, 23]. CHOP expression is crucial for ER stress‐induced apoptosis [23, 24], through transcriptional regulation of the pro‐apoptotic members BCL2 family of proteins [25, 26]. As BCL2 is overexpressed in CLL cells and plays a significant role in their survival, it follows that the UPR and drugs that decrease components of the UPR warrant further investigation in CLL.

In the current study we demonstrate that the ClpP activator, ONC‐212, has significant cytotoxic, cytostatic effects and inhibits the migratory capacity of CLL cells. The data presented suggest that ONC‐212 may represent an effective treatment that warrants further investigation in CLL, particularly for patients with high risk disease.

## 2 MATERIALS AND METHODS

### 2.1 Patient samples

Peripheral blood samples were collected from CLL patients managed at the Royal North Shore Hospital, following informed consent. All patients were diagnosed with CLL according to the international workshop on CLL guidelines [27]. Patient samples are detailed in Table 1. The peripheral blood mononuclear cell (PBMC) fraction from each sample was isolated by Ficoll‐density gradient centrifugation followed by cryopreservation in fetal calf serum (FCS) containing 10% dimethylsulphoxide. PBMC fractions from CLL patients were comprised of >85% CD5^+^/CD19^+^ (CLL) cells, as determined by flow cytometry (data not shown). ATM/TP53 dysfunction and CD38 and ZAP‐70 expression in the patient samples were assessed as described elsewhere [28, 29, 30]. Fluorescent in situ hybridisation (FISH) was performed using the Vysis CLL FISH probe set (Abbot Laboratories, Chicago, IL).

**TABLE 1:** Details of the CLL patient samples studied

CLL patient #	ZAP‐70	CD38	ATM/TP53 function	17p	11q	Treatment history
1	12.06	2.43	N	+/+	+/+	NT
2	2.13	7.70	ND	+/+	+/+	ND
3	19.10	0.30	N	ND	ND	ND
4	8.50	0.10	N	+/+	+/+	FCR
5	26.50	25.05	N	+/+	+/+	FCR, IBR
6	29.20	50.50	ND	+/+	+/+	ND
7	77.40	2.90	3	+/‐ (15)	+/+	FCR
8	4.26	1.49	N	+/+	+/+	NT
9	66.80	84.20	N	+/‐ (12)	+/+	NT
10	5.49	2.43	N	+/‐ (12)	+/+	NT
11	3.30	0.00	1	+/+	+/+	ALEM
12	1.64	0.00	N	+/+	+/+	RCVP/CHOP
13	81.8	0.20	N	+/+	+/+	NT
14	16.11	83.05	N	+/+	+/+	FCR
15	12.79	88.76	N	+/+	+/+	FLAV
16	2.84	4.26	N	+/+	+/+	FCR
17	6.60	15.22	3	ND	ND	FCR
18	1.47	2.22	N	+/+	+/+	FCR
19	1.30	2.40	3	ND	ND	NT

Cutoff values for ZAP‐70 and CD38 expression were 10 and 20%, respectively. ATM/TP53 functional definitions were N ‐ no dysfunction, 1 ‐ *TP53* mutated and 3 ‐ evidence of emerging TP53 dysfunction. FISH results for the 17p (*TP53*) and 11q (*ATM*) loci were defined as +/+ ‐ no evidence of loss and +/‐ heterozygous loss of one copy.

Abbreviations: ALEM, alemtuzumab; FCR, fludarabine, cyclophosphamide, rituximab; FLAV, flavopiridol; IBR, ibrutinib; ND, no data available; NT, no prior treatment; RCVP/CHOP, rituximab, cyclophosphamide, vincristine, prednisolone/cyclophosphamide, doxorubicin, vincristine, prednisolone.

### 2.2 Cell culture

Primary cells were rapidly thawed at 37°C and washed in RPMI 1640 medium (Thermo Fisher Scientific, Waltham, MA) containing 10% FCS, 2 mM L‐glutamine and 1% penicillin/streptomycin (‘complete’ medium). Primary CLL cells were cultured in complete medium, either alone or in co‐culture with a mouse fibroblast cell line expressing the human CD40 ligand (CD40L‐fibroblasts). CD40L‐fibroblasts were seeded at a density of 250 cells/µL a day prior to the introduction of CLL cells. The OSU‐CLL cell line was derived as previously described [31] and obtained from the Human Genetics Sample Bank at Ohio State University under a material transfer agreement. The CD40L‐fibroblasts and the OSU‐CLL and OSU‐CLL‐*TP53ko* cell lines were maintained in complete, RPMI‐1640 medium.

### 2.3 Generation of the OSU‐CLL‐TP53ko line

The *TP53* knock‐out OSU‐CLL (OSU‐CLL‐*TP53*ko) cell line was generated using a doxycycline‐inducible lentiviral CRISPR‐Cas9 technique developed at the Walter and Eliza Hall Institute, Parkville, Victoria, Australia [32]. Knock‐out of the *TP53* gene and the absence of TP53 protein expression were confirmed by direct sequencing and immunoblotting, respectively.

### 2.4 Cell viability assessment

The viability of primary CLL cells or cell lines was determined using the mitochondrial membrane potential dye 1,1′,3,3,3′,3′‐hexamethylindodicarbocyanine iodide (DiIC_1_(5)) and propidium iodide (PI), with analysis by flow cytometry on a LSR Fortessa instrument with DIVA (v8) software (Becton Dickinson, Franklin Lakes, NJ). Cells were incubated for 15 minutes at 37°C with 50 nM DiIC_1_(5) and 30 µg/mL propidium iodide at 37°C. DiIC_1_(5) positive, PI negative cells were considered viable. IC_50_ values were determined using GraphPad Prism software (GraphPad Software, San Diego, CA).

### 2.5 Cell cycle and cell proliferation

For analysis of cell cycle distribution, OSU‐CLL and OSU‐CLL‐*TP53*ko cells were treated with ONC‐212 for 24, 48 or 72 hours. At each time point, cells were harvested, resuspended in 50% ethanol and stored at −20°C for at least 24 hour. Samples were washed in phosphate‐buffered saline (PBS), stained with a solution containing 40 µg/mL PI, 10 mg/mL RNase‐A and 0.1% TritonX‐100 in PBS. The proportion of cells in each cell cycle phase was assessed from the DNA content of the cells, using flow cytometry and ModFit software (Verity Software House, Topsham, ME).

Proliferation of the OSU‐CLL and OSU‐CLL‐*TP53*ko cell lines was investigated by staining cells with the amine dye, carboxyfluorescein succinimidyl ester (CFSE). Cells were stained with 2 µM CFSE for 30 minutes at 37°C and then washed in complete medium. Cells were left untreated or treated with ONC‐212 for 24, 48 or 72 hours. The rate of decay in CFSE fluorescence over time, assessed by flow cytometry, is proportional to the rate of cell proliferation.

### 2.6 Cell migration and assessment of CD49d and CXCR4 expression

The migratory capacity of primary CLL cells was assessed using Transwell permeable supports (MerckSigma, Burlington, MA) with 5 µm pores. CLL patient samples were cultured in complete medium, with or without 2 µM ONC‐212 for 24 hours. Viability was assessed by trypan blue exclusion, and an equal number of viable cells (3 × 10^5^) were loaded into the upper chambers of the Transwell culture inserts. The well below each insert was filled with complete medium, with or without 200 ng/mL SDF‐1α (Peprotech, Rocky Hill, NJ). Following a 3 hours incubation, the medium in the lower chamber was harvested and the number of viable CD5^+^/CD19^+^ cells assessed by flow cytometry.

The effects of ONC‐212 on the expression of the integrin CD49d and the chemokine receptor CXCR4 were assessed using antibodies conjugated to fluorescein isothiocyanate and phycoerythrin, respectively (Biolegend, San Diego, CA). Changes in expression were assessed in terms of a fold‐change relative to untreated controls, which was calculated from the mean fluorescence intensity values.

### 2.7 Immunoblotting

Immunoblotting was performed on patient samples and both OSU‐CLL cell lines. OSU‐CLL and OSU‐CLL‐*TP53*ko cells were treated with 25, 50 or 500 nM ONC‐212 for 24 hours. Primary CLL cells were cultured in medium alone or on a confluent layer of CD40L‐fibroblasts. CLL cells in co‐culture were treated with 0.5, 2.5 or 5 µM ONC‐212 for 24 hours. Cells were then harvested, washed with PBS and lysed in radio‐immuno‐precipitation assay buffer (150 mM sodium chloride, 1.0% Triton X‐100, 0.5% sodium deoxycholate, 0.1% sodium dodecyl sulphate, 50 mM Tris‐HCl, pH 8.0) containing a cocktail of protease and phosphatase inhibitors (MSSafe; Sigma Aldrich, St Louis, MI) for 60 minutes on ice with regular vortexing. Samples were centrifuged and then reducing agent and sample buffer (Life Technologies, Carlsbad, CA) added followed by heating at 70°C for 10 minutes. The proteins were resolved by SDS‐PAGE on 4‐12% BOLT Bis‐Tris pre‐cast gels (Life Technologies) and transferred to polyvinylidene fluoride (PVDF) membranes using the iBlot transfer system (Life Technologies). Non‐specific binding was blocked by incubation with 0.5% milk powder in Tris‐buffered saline with Tween‐20 (TBST) (150 mM NaCl, 20 mM Tris‐HCl, 0.1% Tween‐20, pH 7.4), for 1 hour at room temperature. The membranes were incubated with primary antibodies overnight at 4°C before being washed with TBST and incubated with horse radish peroxide labelled secondary antibodies (Biolegend). Following three washes with TBST, the membranes were incubated in enhanced chemiluminescence substrate (2.5 mM luminol, 0.40 mM p‐Coumaric acid, 100 mM Tris‐HCl pH 8.5 and 0.02% H_2_O_2_) for 90 seconds before imaging on a ChemiDocMP Gel Imaging System (Bio‐Rad Laboratories, Hercules, CA). Relative quantification of the proteins was performed by densitometry using the ImageJ software (https://imagej.net/) with β‐actin as a loading control.

### 2.8 Statistical analyses

All statistical analyses were performed using the T‐test function of GraphPad Prism software.

## 3 RESULTS

### 3.1 ONC‐212 is cytotoxic towards CLL cells in medium alone or in co‐culture with CD40L‐fibroblasts

**FIGURE 1:**
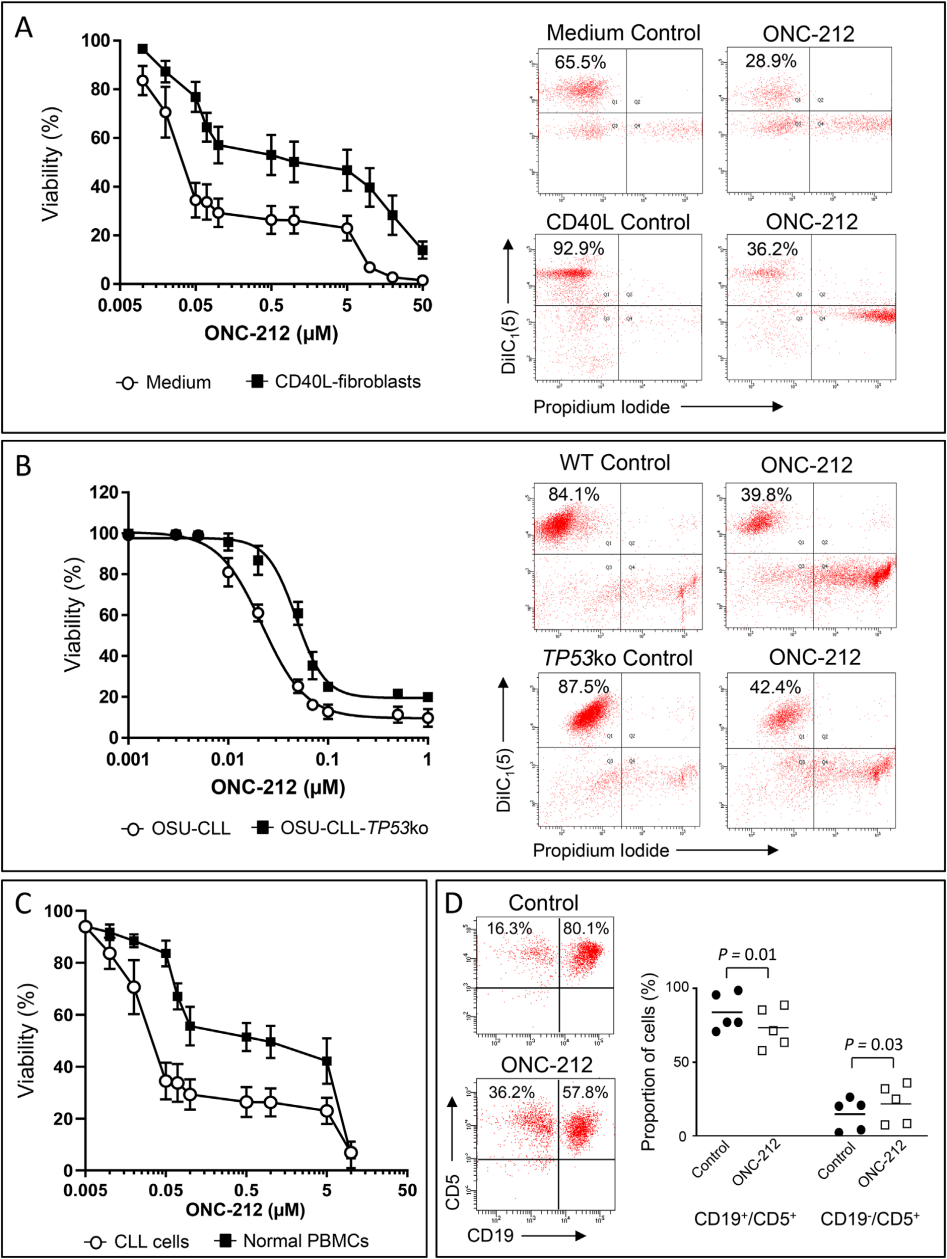
ONC‐212 induced dose‐dependent apoptosis in primary CLL cells and the OSU‐CLL cell line under conditions that mimic the tumour microenvironment and in CLL cells with TP53 lesions. ONC‐212 induced apoptosis in primary CLL cells cultured in medium alone or with fibroblasts (A). ONC‐212 induced dose‐dependent apoptosis in OSU‐CLL and OSU‐CLL‐*TP53*ko cells (B). CLL cells were significantly more sensitive to ONC‐212 than PBMCs from healthy donors (C) and autologous T‐cells (D)

ONC‐212 was cytotoxic in a dose‐dependent manner towards primary CLL cells (n = 10) cultured in medium alone or with CD40L‐fibroblasts (Figure 1A). However, we observed a significant (*P* < .001) difference in the IC_50_ for ONC‐212 under the two culture conditions; the IC_50_ values were 33.7 +/‐ 11.9 nM and 404 +/‐ 70.6 nM against cells in medium or with CD40L‐fibroblasts, respectively. Importantly, we observed no difference in the sensitivity of CLL cells from patients with *TP53* lesions (n = 4) to ONC‐212.

### 3.2 ONC‐212 is cytotoxic against OSU‐CLL and OSU‐CLL‐TP53ko cells

To explore the effects of ONC‐212 against TP53 deficient CLL cells, we generated an OSU‐CLL cell line in which *TP53* was deleted using the CRISPR‐Cas9 technology. ONC‐212 was cytotoxic against OSU‐CLL and OSU‐CLL‐*TP53*ko cells in a dose dependent manner (Figure 1B). The IC_50_ values for ONC‐212 in these cell lines were 21.90 +/− 1.70 nM and 48.0 +/− 3.31 nM (*P* = .007) for the OSU‐CLL and OSU‐CLL‐*TP53*ko lines, respectively. OSU‐CLL cells transfected with CRISPR‐Cas9 and the *TP53* guide RNA, but not treated with doxorubicin, were examined as a control for the transfection procedures involved. No significant difference (*P* = .28) in the sensitivity of these cells and OSU‐CLL cells to ONC‐212 was observed (Figure S1A), confirming that the reduced sensitivity of the OSU‐CLL‐*TP53*ko cells to ONC‐212 is a result of the *TP53* deletion.

### 3.3 ONC‐212 is selectively more toxic towards CLL cells than normal PBMCs and T‐cells

Dose response analyses comparing the effects of ONC‐212 against PBMCs from healthy individuals (n = 4) and CLL cells (n = 4) cultured in medium alone demonstrated that CLL cells were significantly (*P* < .005) more sensitive to the drug than PBMCs from healthy individuals (Figure 1C). The IC_50_ values for ONC‐212 were 33.7 +/− 11.9 nM and 537.4 +/− 18.7 nM, against CLL cells and PBMCs from healthy individuals, respectively.

The effects of ONC‐212 against CLL (CD19^+^/CD5^+^) and T‐cells (CD19^−^/CD5^+^) in PBMC fractions from CLL patients were assessed by examining the proportions of these cells before and after treatment with 2 µM ONC‐212 for 48 hours. The decrease in the proportion of viable CLL cells and concomitant increase in the proportion of viable T‐cells following treatment, suggest that ONC‐212 is more cytotoxic towards CLL cells than T‐cells in CLL patient PBMC fractions (Figure 1D).

### 3.4 ONC‐212 induces G0/G1 cell cycle arrest and inhibits the proliferation of OSU‐CLL and OSU‐CLL‐TP53ko cells

**FIGURE 2:**
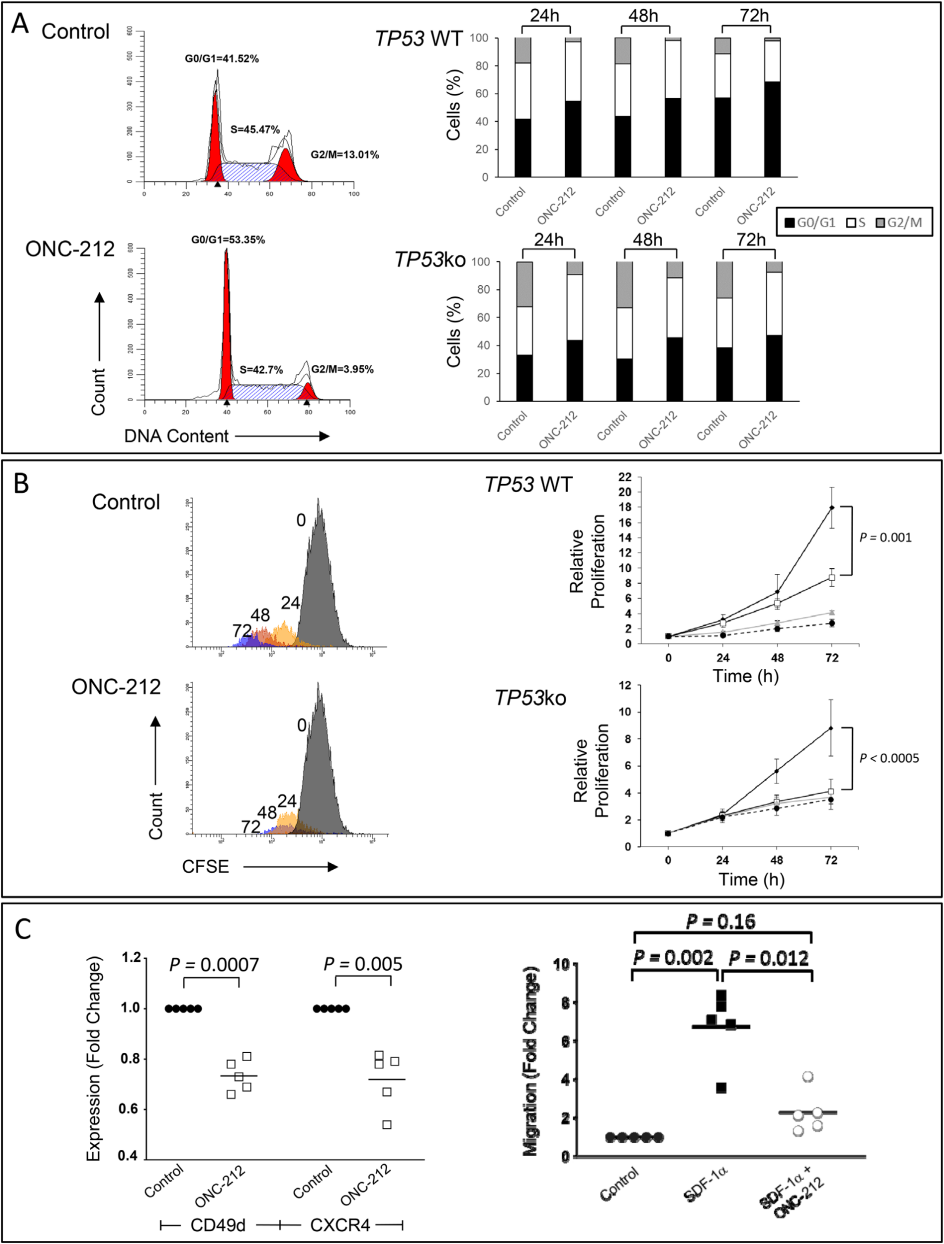
ONC‐212 treatment arrested the cell cycle progression, reduced the proliferation, and attenuated the migration of CLL cells. ONC‐212 treatment resulted in an accumulation of OSU‐CLL and OSU‐CLL‐TP53ko cells in G0/G1 with a concomitant decrease in the proportion of cells in G2/M and S‐phase (A). ONC‐212 also significantly reduced the rate of proliferation of the OSU‐CLL and OSU‐CLL‐*TP53*ko cells over a 72 h time course (B). Expression of the integrin CD49d and the chemokine receptor CXCR4 was significantly lower on primary CLL cells following treatment with ONC‐212. Consistent with the decrease in CXCR4 expression, ONC‐212 treatment also resulted in a significant decrease in the migratory capacity of primary CLL cells towards the CXCR4 ligand, SDF‐1α (C)

ONC‐212 induced a significant (*P* < .05) accumulation of cells from both the OSU‐CLL and OSU‐CLL‐*TP53*ko lines in G0/G1, with a concomitant decrease in the proportion of cells in G2/M at each of the three time points (*P* < .05; Figure 2A). In the *TP53*ko cells, a significant (*P* < .05) increase in the proportion of cells in S phase was also observed with ONC‐212 treatment at each time point.

Consistent with the effects of the drug on the cell cycle, we observed a significant effect of ONC‐212 on the proliferation rate of both the OSU‐CLL and OSU‐CLL‐*TP53*ko cells (Figure 2B). ONC‐212 at its IC_25_, IC_50_ and IC_75_ concentrations against the lines induced a significant (*P* < .05) decrease in the rate of proliferation of both lines at 48 and 72 hours, relative to untreated control cells.

### 3.5 ONC‐212 restricts the adhesive and migratory capacities of CLL cells

ONC‐212 treatment (2 µM for 24 hours) of primary CLL cells resulted in a significant (*P* < .01) down‐regulation of expression of the chemokine receptor CXCR4 and integrin CD49d, relative to cells cultured in medium alone (Figure 2C, left). ONC‐212 had no significant effect on the expression of CD49d in healthy B‐lymphocytes but, contrary to the effect in CLL cells, significantly (*P* = .035) increased the expression of CXCR4 (Figure S2, left). To investigate the functional consequence of decreased CXCR4 expression in CLL cells, we assessed the capacity of primary CLL cells and healthy B‐cells to migrate through a permeable support towards the CXCR4 ligand, stroma‐derived factor 1α (SDF‐1α). An equal number of viable cells were loaded into the upper chambers of Transwell inserts, and the number of viable cells that migrated through the support towards SDF‐1α was assessed after 3 hours by flow cytometry. The presence of SDF‐1α significantly (*P* = .002) increased the migration of viable CLL and healthy B‐cells, relative to the migration of cells cultured in medium alone (Figure 2C, right and Figure S2, right). Consistent with the effects of ONC‐212 on CXCR4 expression, pre‐treatment of the CLL cells with ONC‐212 significantly (*P* = .012) reduced the number of viable CLL cells that migrated through the permeable support towards SDF‐1α. In contrast, ONC‐212 had no significant effect on the migration of primary healthy B‐cells (Figure S2, right).

### 3.6 ONC‐212 increases expression of CIpP and alters the expression of components of the UPR in CLL cells

**FIGURE 3:**
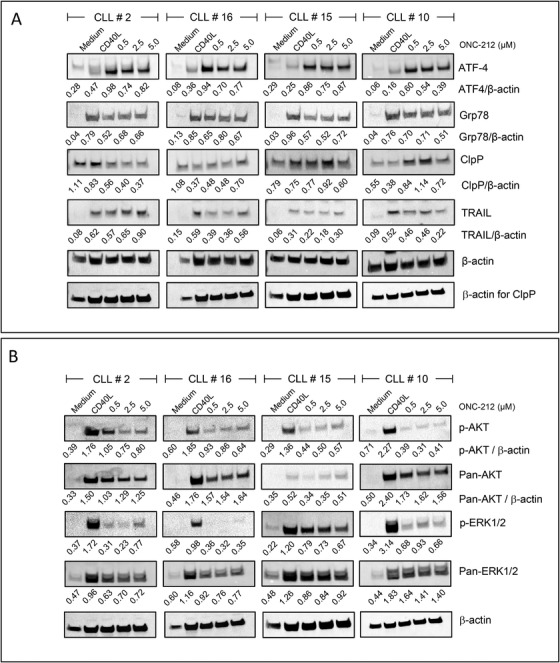
The cytotoxicity of ONC‐212 towards CLL cells in co‐culture with stromal cells is associated with changes in expression of proteins involved in the UPR and signaling downstream of the B‐cell receptor. CLL cells were cultured in medium alone (Medium) or in contact with CD40L‐fibroblasts (CD40L). CD40L‐fibroblast co‐cultured cells were treated with the indicated doses of ONC‐212 for 24 hour. The CLL # above each series of immunoblots indicates the individual patient sample analysed, which corresponds to the patients detailed in Table 1. ONC‐212 induced expression of ATF4 and CIpP and decreased expression of Grp78 in CLL patient samples (n = 4) co‐cultured with CD40L‐fibroblasts (A). Phosphorylation of the AKT and ERK1/2 proteins induced in CLL cells by co‐culture with CD40L‐fibroblasts, was decreased in CLL cells treated with the indicated doses of ONC‐212 (B)

Next, we investigated the mechanisms of action of ONC‐212 in CLL cells by examining changes in the expression of the mitochondrial protease CIpP, ATF4 and Grp78, and the tumour necrosis factor‐related apoptosis‐inducing ligand (TRAIL), by immunoblotting. PBMC fractions from four CLL patients were cultured in medium alone or in contact with CD40L fibroblasts. CLL cells in co‐culture with stromal cells were either left untreated or were treated with the indicated doses of ONC‐212 for 24 hours. Stromal co‐culture increased expression of ATF4, Grp78 and TRAIL and decreased expression of CIpP in all 4 CLL samples assessed (Figure 3A). In response to treatment with ONC‐212 at the doses indicated, we observed increased expression of ATF4 and decreased expression of Grp78 at all doses in all four patient samples. The effects of ONC‐212 on expression of CIpP and TRAIL were more variable but in three of the four samples (CLL #’s 16, 15 and 10), increased expression of CIpP and decreased expression of TRAIL were observed following treatment with at least one dose of ONC‐212.

### 3.7 The cytotoxic effects of ONC‐212 are consistent with inhibition of BCR signaling and a pro‐apoptotic shift in the balance of BCL2 family proteins

The effects of ONC‐212 on signaling downstream of the BCR and on expression of BCL‐2 family proteins were explored by immunoblotting. Co‐culture of primary CLL cells with CD40L‐fibroblasts increased expression of total (two/three samples) and phosphorylated AKT and ERK1/2 protein (three/three samples) (Figure 3B). Treatment of the CLL samples with ONC‐212 while in co‐culture with stromal cells resulted in a significant attenuation of the stroma‐induced phosphorylation of both AKT and ERK1/2‐MAPK.

In the OSU‐CLL and OSU‐CLL‐*TP53*ko cells, ONC‐212 reduced the phosphorylation and total expression of ERK1/2 (Figure S2B). However, the drug had opposing effects on AKT phosphorylation in the OSU‐CLL and OSU‐CLL*TP53*ko lines; in contrast to the primary samples and WT OSU‐CLL line, increased levels of AKT phosphorylation were observed in the *TP53*ko line following ONC‐212 treatment (Figure S2B).

**FIGURE 4:**
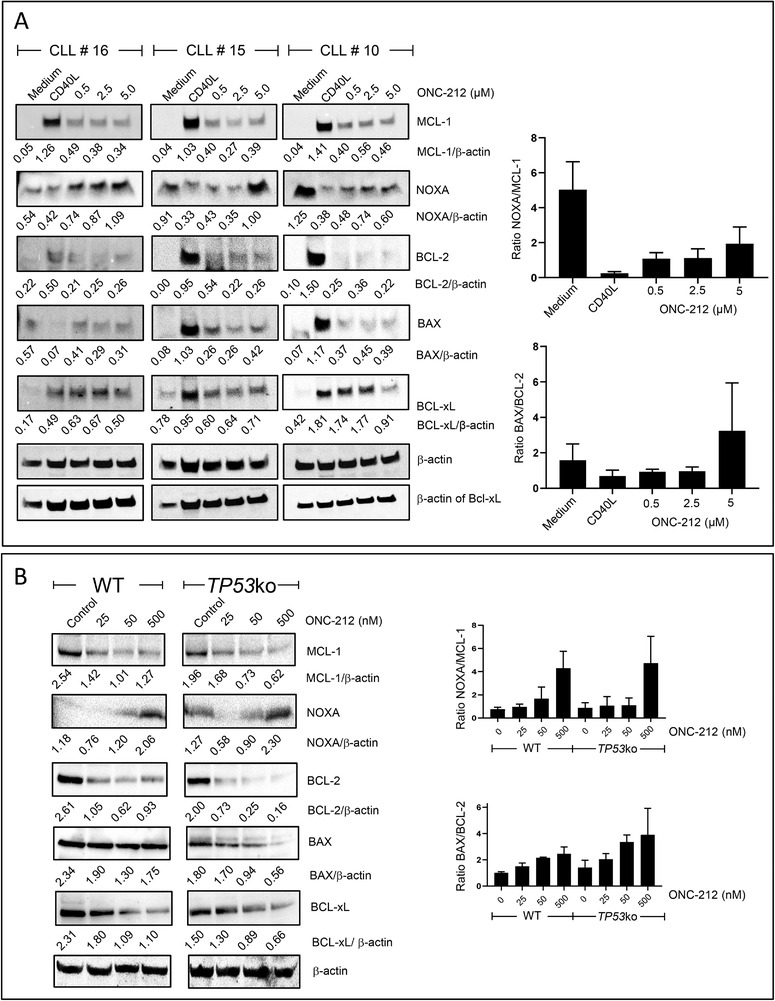
ONC‐212 induces a pro‐apoptotic shift in the balance of proteins of the BCL2 family. ONC‐212 reduced expression of MCL‐1, BCL‐2 and BCL‐xL and increased the expression of NOXA and BAX in primary CLL patient samples (n = 4) co‐cultured with CD40L‐fibroblasts (A) and in both the OSU‐CLL and OSU‐CLL‐*TP53*ko cell lines (B). The CLL # above each series of immunoblots indicates the patient sample analysed, which corresponds to the patient samples detailed in Table 1. A representative image from three replicates is shown in panel B

Next, we examined the effects of ONC‐212 on the expression of the BCL2 family members, MCL1, BCL2, BCLxL, NOXA and BAX. NOXA and BAX are negative regulators of MCL1 and BCL2, respectively, so ratios of these proteins were calculated to assess pro and anti‐apoptotic shifts in the balance of these proteins. Stromal co‐culture of primary CLL cells resulted in up‐regulation of MCL1, BCL2 and BCLxL, with concomitant decreased expression of NOXA, BAX and PUMA (in at least two of the three samples analysed), compared to cells cultured in medium alone (Figure 4A). ONC‐212 treatment reduced the expression of MCL1, BCL2 and BCLxL and increased the expression of NOXA and BAX. These changes resulted in a significant increase in both the NOXA/MCL1 and BAX/BCL2 ratios, shown in the histograms (Figure 4A). Similar changes in expression and hence ratios of the BCL2 family proteins were observed in both OSU‐CLL and OSU‐CLL‐*TP53*ko cells following treatment with ONC‐212 (Figure 4B). Collectively, these data suggest that ONC‐212 induces a pro‐apoptotic shift in the balance of the MCL1, BCL2 and BCLxL proteins in CLL cells and demonstrate that these effects are independent of TP53 status.

## 4 DISCUSSION

Clinical trials of ibrutinib, idelalisib and venetoclax have revolutionised the treatment of CLL. Despite high response rates, a significant proportion of patients treated with these targeted agents, relapse or develop an aggressive transformation of their disease. While loss or mutation of *TP53* remains an indication of poor response and survival rates [33], recent studies have shown that mutations in genes, including *BTK*, *PLC‐γ2* and *BCL2*, are frequently acquired during treatment and are associated with drug resistance [34]. This highlights the need for continued research into novel treatment approaches, particularly for patients with high risk or relapsed disease.

In the current study we demonstrate that the imipridone, ONC‐212, has significant activity against primary CLL cells under in vitro conditions that mimic the TME and against a *TP53*‐deficient CLL cell line. However, as observed with other drugs, including fludarabine [35, 36] and venetoclax [37], co‐culture of primary CLL cells with stromal cells and *TP53*ko were associated with a significant reduction in the sensitivity of the leukemic cells to ONC‐212 (Figure 3). Although this suggests that higher doses of ONC‐212 would be required to overcome the effects of the TME, the IC50 for ONC‐212 against healthy B‐cells (537.4 nM) was higher than against CLL cells cultured with CD40L‐fibroblasts (404 nM), suggesting a therapeutic window may be likely.

The reduced sensitivity of *TP53*ko CLL cells to ONC‐212 (Figure 1B) may be related to the increased phosphorylation of AKT we observed in the *TP53*ko cells following ONC‐212 treatment (Figure S2B). Despite their reduced sensitivity, the IC50 for ONC‐212 against *TP53*ko CLL cells was still in the nanomolar range, which is consistent with previous studies demonstrating the efficacy of the imipridones against cancers harbouring mutations of *TP53* [38]. The reduced sensitivity of the OSU‐CLL*TP53*ko cells, but not primary CLL cells with TP53 lesions, to ONC‐212 may be due to differences in the clonal frequency of the *TP53* lesions between the cells. In the cell line, approximately 100% of the cells are TP53 deficient, while in the three patient samples with deletions of *TP53* the frequency of deletion was <20%. In the three samples with TP53 dysfunction but no deletion, it was not possible to ascertain the size of the dysfunctional clone. Therefore, in the patient samples any difference in the sensitivity of the sub‐clonal populations to ONC‐212 may be masked by the predominant clone with no TP53 lesion.

The effects we observed of ONC‐212 on PI3‐kinase and MAPK‐ERK1/2 signaling and on the BCL2 family of proteins are consistent with previous studies of ONC‐201 [39] and raise the possibility that the drug may be effective for CLL patients with ibrutinib or venetoclax resistant disease or may reduce the risk of disease evolution and development of drug resistant clones. The sensitivity of primary CLL cells to the cytotoxic effects of ONC‐212, compared to PBMC fractions from healthy individuals or T‐cells from CLL patients (Figures 1C and 1D), suggests that ONC‐212 may also specifically target the leukemic cells, in a manner similar to venetoclax and ibrutinib [40, 41, 42], due to the dependence of the CLL cells on signaling down‐stream of the BCR and on the BCL2‐family proteins. Interestingly, ONC‐212 also had distinct effects on healthy B‐cells and CLL cells, in terms of CD49d and CXCR4 expression and SDF‐1α‐induced migration. ONC‐212 treatment of healthy B‐cells had no effect on CD49d expression or on cell migration but significantly increased the expression of CXCR4 (Figure S2), suggesting another way in which ONC‐212 may specifically target CLL cells.

Studies of BCR‐targeted therapies, including ibrutinib, highlight the importance of restricting the interaction between CLL cells and the ‘accessory’ cells that comprise the TME. The lymphocytosis that often occurs in patients treated with ibrutinib and the durability of the response depend on the liberation of CLL cells from the lymph nodes and marrow and restricting the leukemic cells from re‐populating these tissues. These effects are, at least in part, mediated by downregulation of expression of the integrin CD49d [43, 44] and the chemokine receptor CXCR4 [45]. The reduced expression of CD49d and CXCR4 and inhibition of CLL‐cell migration under the influence of SDF‐1α (Figure 2C) following ONC‐212 treatment suggest that, similar to the BCR‐targeted agents, ONC‐212 may reduce the migratory capacity of CLL cells and prevent their retention within the lymph nodes. Previous studies on imipridones also suggest that the G‐coupled protein receptor, CXCR4, may be targeted by ONC‐212 [10], however the current study does not confirm or exclude the possibility that CXCR4 is a direct target of ONC‐212.

This is the first study to suggest that the mitochondrial protease, CIpP, plays a role in the response of CLL cells to cytotoxic drug treatment. Recent studies demonstrate that activation of CIpP is the principal mechanism of action of ONC‐201 and ONC‐212, against a range of different cancers [11]. Interestingly, our observation that CIpP expression was lower in untreated *TP53*ko than WT OSU‐CLL cells, suggests that CIpP may be regulated in a TP53‐dependent manner and may contribute to the reduced sensitivity of *TP53*ko cells to ONC‐212.

ONC‐212 also had effects on the expression of ATF4, Grp78, CIpP and TRAIL in CLL cells (Figure 3A and Figure S3A). These changes are not only indicative of the mechanisms of action of the drug but also reinforce the notion that the UPR plays an important role in CLL‐cell survival. ATF4 is overexpressed in many forms of cancer [46] and plays a key role in the adaption of tumour cells to the stress of rapid proliferation, nutrient and oxygen deprivation and the accumulation of misfolded proteins. ATF4 can promote cell survival but under certain conditions, such as prolonged stress, can also trigger apoptosis [47, 48, 2, 49]. The pro‐apoptotic functions of ATF4 include suppression of the pro‐survival BCL2‐family proteins through increased activity of the transcription factor CHOP and the subsequent increase in expression of pro‐apoptotic members of the BCL2 family [48]. Our data showing that increased ATF4 expression is inversely associated with expression of BCL2, MCL1 and BCLxL (Figures 3 and 4 and Figure S2A) support the notion that increased ATF4 expression is associated with a pro‐apoptotic shift in expression of the BCL2‐family proteins in CLL cells and increased cell death (Figure 1A). Increased ATF4 expression may also be involved in the effects we observed of ONC‐212 on cell cycle phase distribution of the OSU‐CLL cells. These data suggest that, in addition to the cytotoxic effects of ONC‐212, the drug also has significant cytostatic effects on CLL cells (Figures 2A and 2B). Accumulation of cells in G0/G1 was concomitant with increased ATF4 expression, which is consistent with the role of ATF4 in regulating p21 expression [50].

The current study may appear to contradict that of [19] who showed that BCR signaling was associated with activation of the UPR [19]. However, it is apparent that UPR activation may induce either survival or apoptosis, depending on the cell type and context [51, 52]. Despite other studies showing that inhibitors of the UPR increase TRAIL‐mediated cell death [53], overall we observed a relatively minor decrease in TRAIL expression in three of the four CLL patient samples following treatment with ONC‐212 (Figure 3A). While CLL cells are reported to be inherently resistant to TRAIL‐mediated cell death [54], studies also suggest that CLL cells can be sensitized to TRAIL‐induced apoptosis under certain conditions [55]. Although no marked changes in TRAIL expression were observed, further studies examining the functionality of this pathway could be conducted to determine whether the drug modulates the sensitivity of CLL cells to TRAIL‐mediated cell death.

The glucose‐regulated family of genes (Grp), which include Grp78, acts as chaperones at the ER and is upregulated in response to accumulation of misfolded proteins [56]. The increase in Grp78 expression observed in primary CLL cells co‐cultured with stromal cells (Figure 3A) supports the notion that Grp78 is involved in the adaption of CLL cells to conditions that support their survival. As these in vitro conditions mimic aspects of the lymph node microenvironment, it is conceivable that CLL cells within the lymph nodes may also express high levels of Grp78 as part of their adaption to conditions that drive rapid proliferation. The importance of Grp78 has been identified in different forms of leukemia, including CLL [57, 58]. In a mouse model, Grp78 knock‐out suppressed the activation of the AKT/mTOR pathway and the accumulation of leukemic blasts [59], suggesting that Grp78 may play an important role in leukemia progression. In other cancers, Grp78 knock‐down has also been shown to enhance apoptosis via a mechanism that involves reduced AKT activity [60]. It is also conceivable that the crosstalk between Grp78 and AKT signaling demonstrated in the study by Wey et al, may explain the decreased phosphorylation of AKT we observed with ONC‐212 (Figures 4A and 4B).

In conclusion, the data presented in the current study suggest that ONC‐212 may represent a novel therapeutic option for patients with CLL. The efficacy of ONC‐212 under conditions that mimic the TME and against TP53 dysfunctional CLL cells raises the possibility that this treatment approach may be particularly effective in patients with a poor prognosis or for patients who develop disease resistant to BCR or BCL2‐targeted therapies.

## CONFLICT OF INTEREST

Edwin J. Iwanowicz and Henk Lang have financial interests in Madera Therapeutics, LLC.  In addition, Donald S. Karanewsky is a consultant for Madera Therapeutics, LLC.

## AUTHOR CONTRIBUTIONS

Narjis Fatima, Yandong Shen, Kyle Crassini and Oliver G. Best designed and performed experiments, analysed the data and assisted in preparation of the manuscript. Edwin J. Iwanowicz, Henk Lang and Donald S. Karanewsky provided ONC‐212 and assisted in preparation of the manuscript. Richard I. Christopherson and Stephen P. Mulligan designed experiments and assisted in the preparation of the manuscript.

## Supporting information

FIGURE S1 Transfection of OSU‐CLL cells with Cas9 and *TP53* guide RNA has no effect on the sensitivity of the cells to ONC‐212.
OSU‐CLL cells transfected with Cas9 and the *TP53* guide RNA but not treated with doxycycline were treated with a range of doses of ONC‐212. No significant difference in the IC_50_ values for ONC‐212 was observed between these control cells and wild‐type (WT) OSU‐CLL cells

FIGURE S2 ONC‐212 increased CXCR4 expression but had no effect on SDF‐1a‐induced cell migration in healthy B‐cells.
Healthy B‐cells were treated cultured in medium alone with or without treatment with 2µM ONC‐212 for 24 hours. Expression of CD49d and CXCR4 and the number of viable cells migrating across a permeable support towards SDF‐1α were assessed by flow cytometry. ONC‐212 had no effect on CD49d expression but significantly increased expression of CXCR4 (left). ONC‐212 treatment had no effect on the number of healthy B‐cells that migrated across the permeable support under the influence of SDF‐1α (right)

FIGURE S3 ONC‐212 induced protein changes consistent with activation of CIpP and inhibition of the UPR and BCR signaling in OSU‐CLL and OSU‐CLL‐*TP53*ko cells. Treatment of OSU‐CLL and OSU‐CLL‐TP53ko cells with ONC‐212 induced expression of ATF4, CIpP and TRAIL and decreased expression of Grp78 in both cell lines (A). ONC‐212 reduced the phosphorylation of ERK1/2‐MAPK in both the *TP53* WT and knock‐out OSU‐CLL cells. Phosphorylation of AKT was reduced by ONC‐212 treatment in WT, but not *TP53*‐ko, OSU‐CLL cells (B)

## Data Availability

The data that support the findings of this study are available from the corresponding author upon reasonable request.
