# Supplementary material for: The ClpP activator ONC‐212 (TR‐31) inhibits BCL2 and B‐cell receptor signaling in CLL
Source: EJHaem. 2021 Jan 14;2(1):81–93. doi: 10.1002/jha2.160 (PMC9175891; doi:10.1002/jha2.160)
Supplement: Supplementary file 1 — FIGURE S1 Transfection of OSU‐CLL cells with Cas9 and TP53 guide RNA has no effect on the sensitivity of the cells to ONC‐212. OSU‐CLL cells transfected with Cas9 and the TP53 guide RNA but not treated with doxycycline were treated with a range of doses of ONC‐212. No significant difference in the IC50 values for ONC‐212 was observed between these control cells and wild‐type (WT) OSU‐CLL cells [file JHA2-2-81-s002.pptx]

## Slide 1
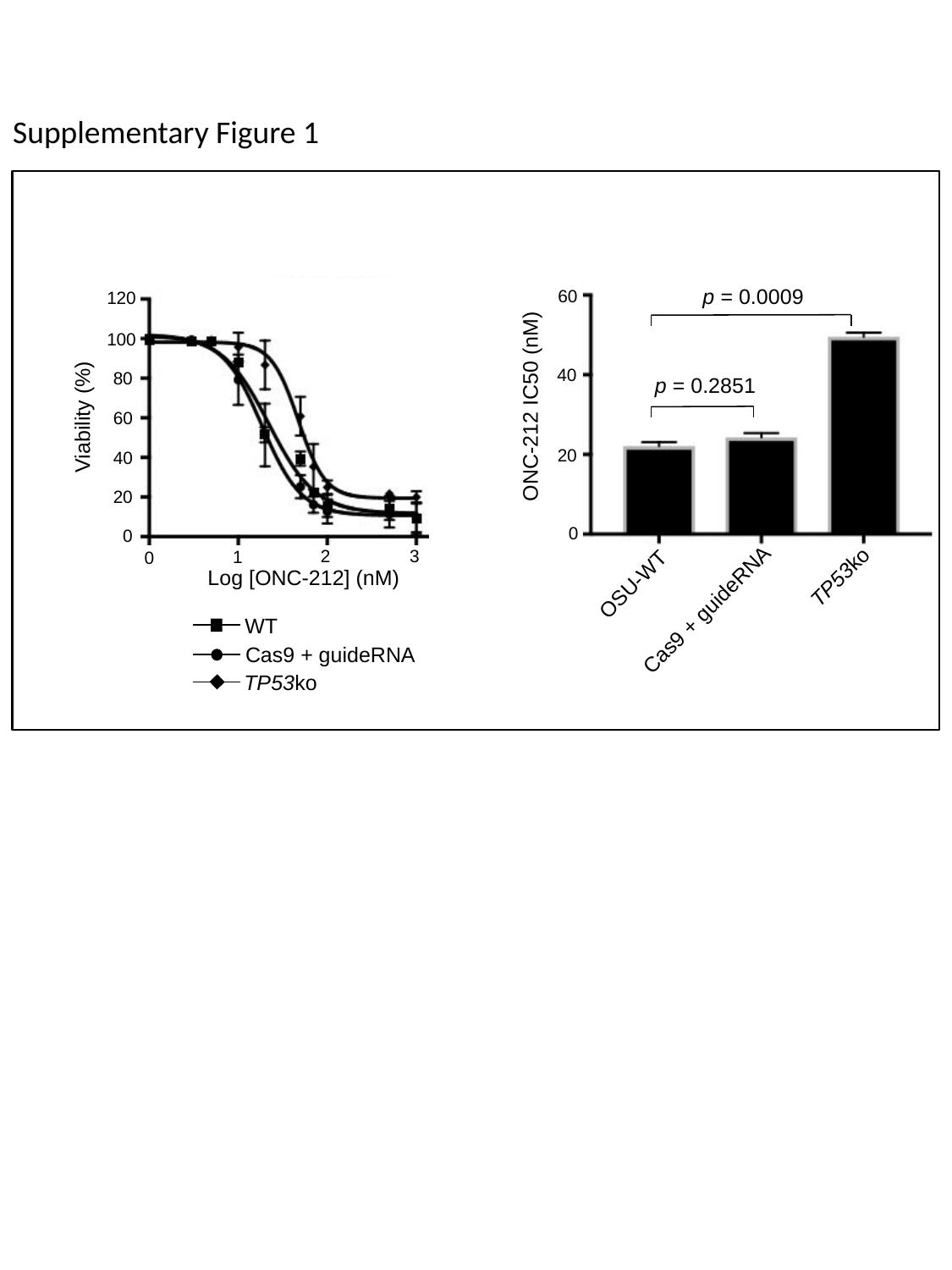

Supplementary Figure 1
p = 0.0009
60
120
***
100
40
80
p = 0.2851
ONC-212 IC50 (nM)
Viability (%)
60
20
40
20
0
0
2
3
1
0
TP53ko
Log [ONC-212] (nM)
OSU-WT
Cas9 + guideRNA
WT
Cas9 + guideRNA
TP53ko
