# Supplementary material for: The ClpP activator ONC‐212 (TR‐31) inhibits BCL2 and B‐cell receptor signaling in CLL
Source: EJHaem. 2021 Jan 14;2(1):81–93. doi: 10.1002/jha2.160 (PMC9175891; doi:10.1002/jha2.160)
Supplement: Supplementary file 2 — FIGURE S2 ONC‐212 increased CXCR4 expression but had no effect on SDF‐1a‐induced cell migration in healthy B‐cells. Healthy B‐cells were treated cultured in medium alone with or without treatment with 2µM ONC‐212 for 24 hours. Expression of CD49d and CXCR4 and the number of viable cells migrating across a permeable support towards SDF‐1α were assessed by flow cytometry. ONC‐212 had no effect on CD49d expression but significantly increased expression of CXCR4 (left). ONC‐212 treatment had no effect on the number of healthy B‐cells that migrated across the permeable support under the influence of SDF‐1α (right) [file JHA2-2-81-s001.pptx]

## Slide 1
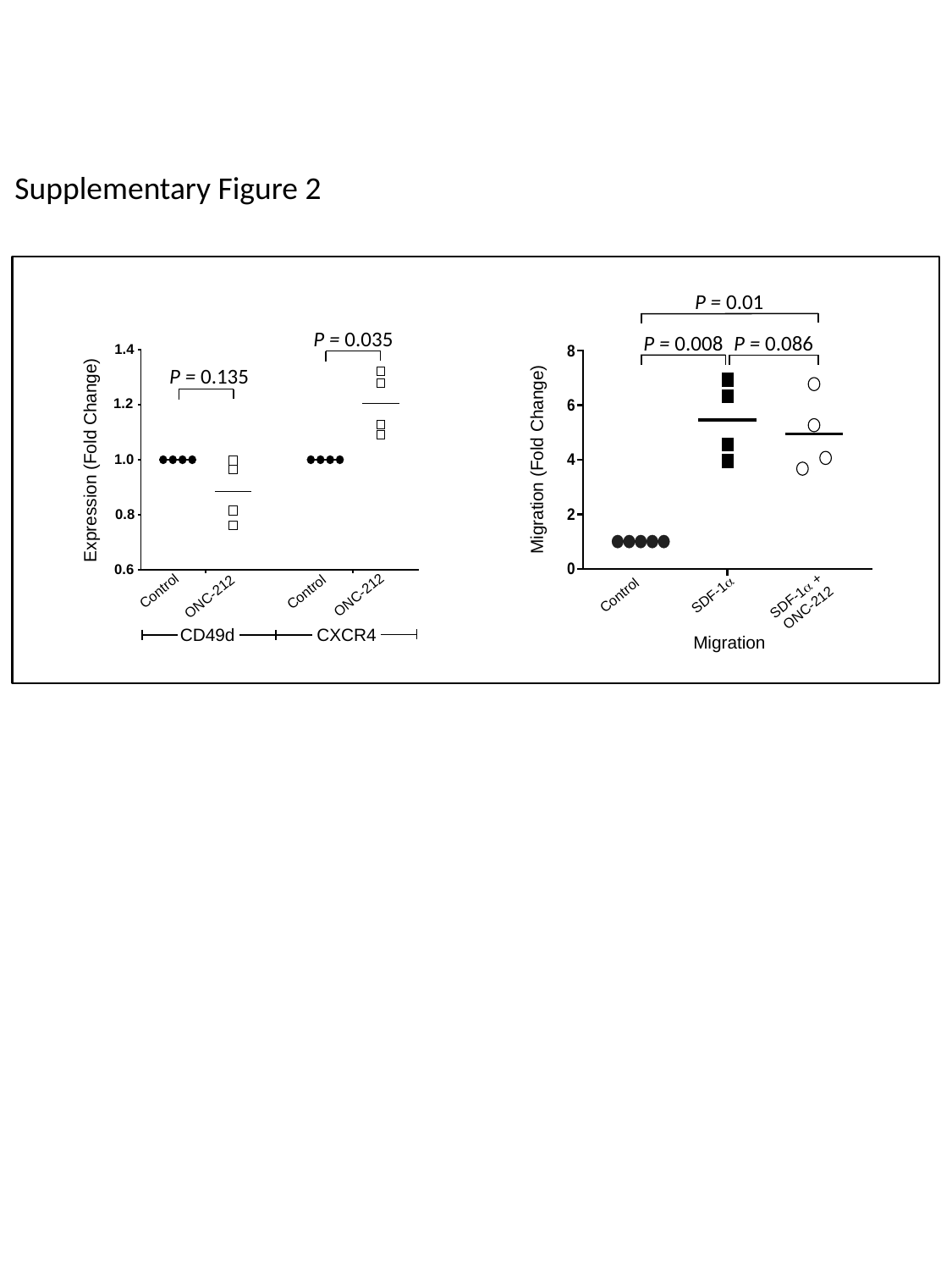

Supplementary Figure 2
P = 0.01
P = 0.035
P = 0.086
P = 0.008
1.4
P = 0.135
1.2
Migration (Fold Change)
Expression (Fold Change)
1.0
0.8
0.6
Control
Control
Control
SDF-1a +
ONC-212
SDF-1a
ONC-212
ONC-212
CD49d
CXCR4
Migration
