# Supplementary material for: The ClpP activator ONC‐212 (TR‐31) inhibits BCL2 and B‐cell receptor signaling in CLL
Source: EJHaem. 2021 Jan 14;2(1):81–93. doi: 10.1002/jha2.160 (PMC9175891; doi:10.1002/jha2.160)
Supplement: Supplementary file 3 — FIGURE S3 ONC‐212 induced protein changes consistent with activation of CIpP and inhibition of the UPR and BCR signaling in OSU‐CLL and OSU‐CLL‐TP53ko cells. Treatment of OSU‐CLL and OSU‐CLL‐TP53ko cells with ONC‐212 induced expression of ATF4, CIpP and TRAIL and decreased expression of Grp78 in both cell lines (A). ONC‐212 reduced the phosphorylation of ERK1/2‐MAPK in both the TP53 WT and knock‐out OSU‐CLL cells. Phosphorylation of AKT was reduced by ONC‐212 treatment in WT, but not TP53‐ko, OSU‐CLL cells (B) [file JHA2-2-81-s003.pptx]

## Slide 1
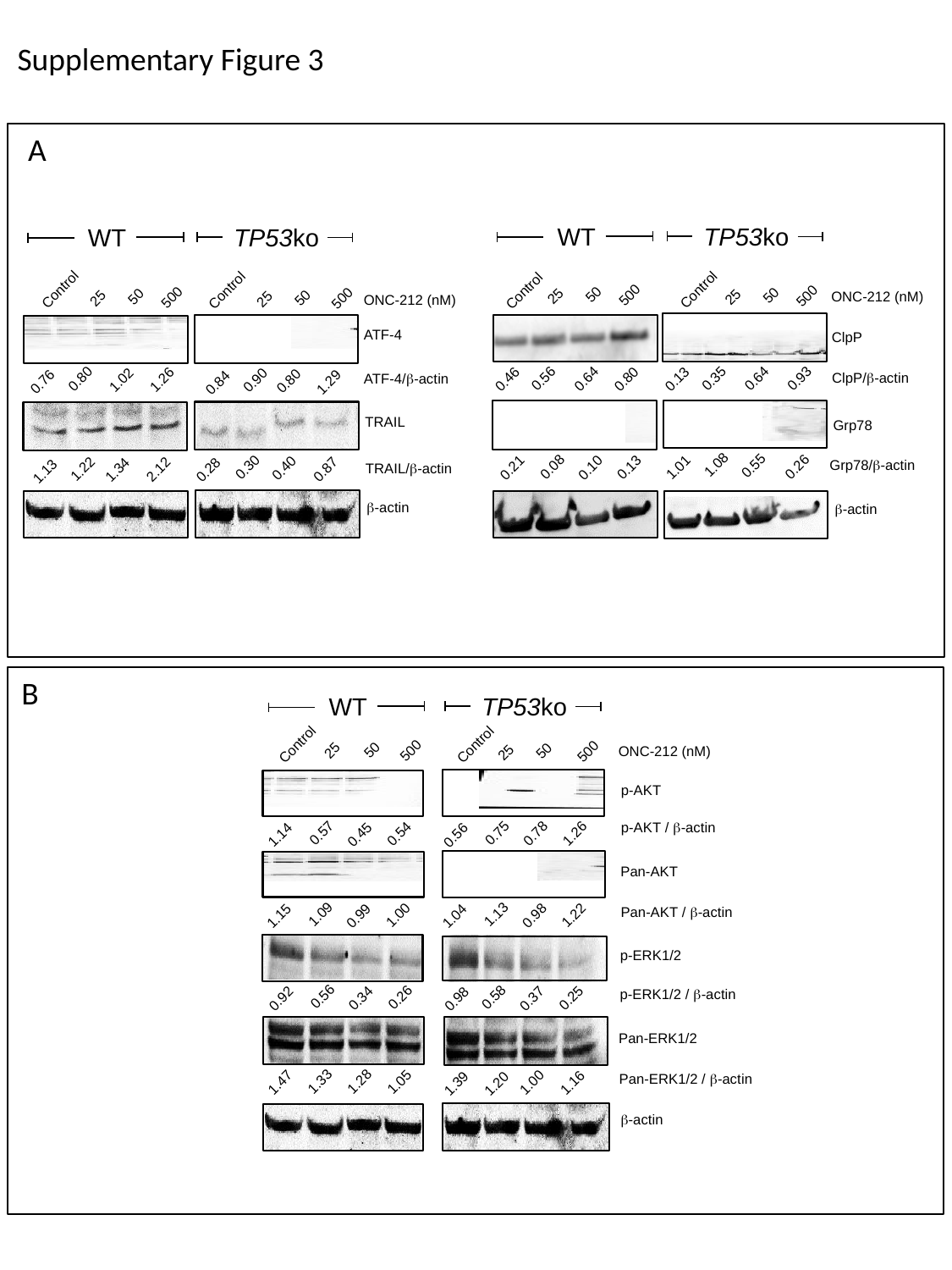

Supplementary Figure 3
A
TP53ko
WT
TP53ko
WT
Control
Control
Control
Control
50
500
50
500
25
ONC-212 (nM)
50
25
500
50
500
25
25
ONC-212 (nM)
ATF-4
ClpP
ClpP/b-actin
0.35
0.93
0.64
0.56
0.64
0.80
0.13
0.80
0.46
ATF-4/b-actin
1.26
1.02
0.90
0.80
0.76
1.29
0.84
TRAIL
Grp78
1.08
Grp78/b-actin
0.55
0.26
0.08
0.30
1.01
0.13
0.10
0.21
0.40
TRAIL/b-actin
1.22
0.87
2.12
1.34
0.28
1.13
b-actin
b-actin
B
TP53ko
WT
Control
Control
50
25
500
50
ONC-212 (nM)
500
25
p-AKT
p-AKT / b-actin
0.57
0.75
1.26
0.78
0.54
0.45
1.14
0.56
Pan-AKT
Pan-AKT / b-actin
1.09
1.13
1.00
1.22
0.98
0.99
1.15
1.04
p-ERK1/2
p-ERK1/2 / b-actin
0.56
0.58
0.26
0.25
0.34
0.92
0.37
0.98
Pan-ERK1/2
Pan-ERK1/2 / b-actin
1.33
1.28
1.05
1.00
1.47
1.16
1.39
1.20
b-actin
